# Supplementary material for: A Global Screen for Assembly State Changes of the Mitotic Proteome by SEC-SWATH-MS
Source: Cell Syst. 2020 Feb 26;10(2):133–155.e6. doi: 10.1016/j.cels.2020.01.001 (PMC7042714; doi:10.1016/j.cels.2020.01.001)

Q16539 | MK14_HUMAN | MAPK14 CSBP CSBP1 CSBP2 CSPB1 MXI2 SAPK2A
Monomer MW [kDa]: 41.293 Monomer expected elution fraction: 48

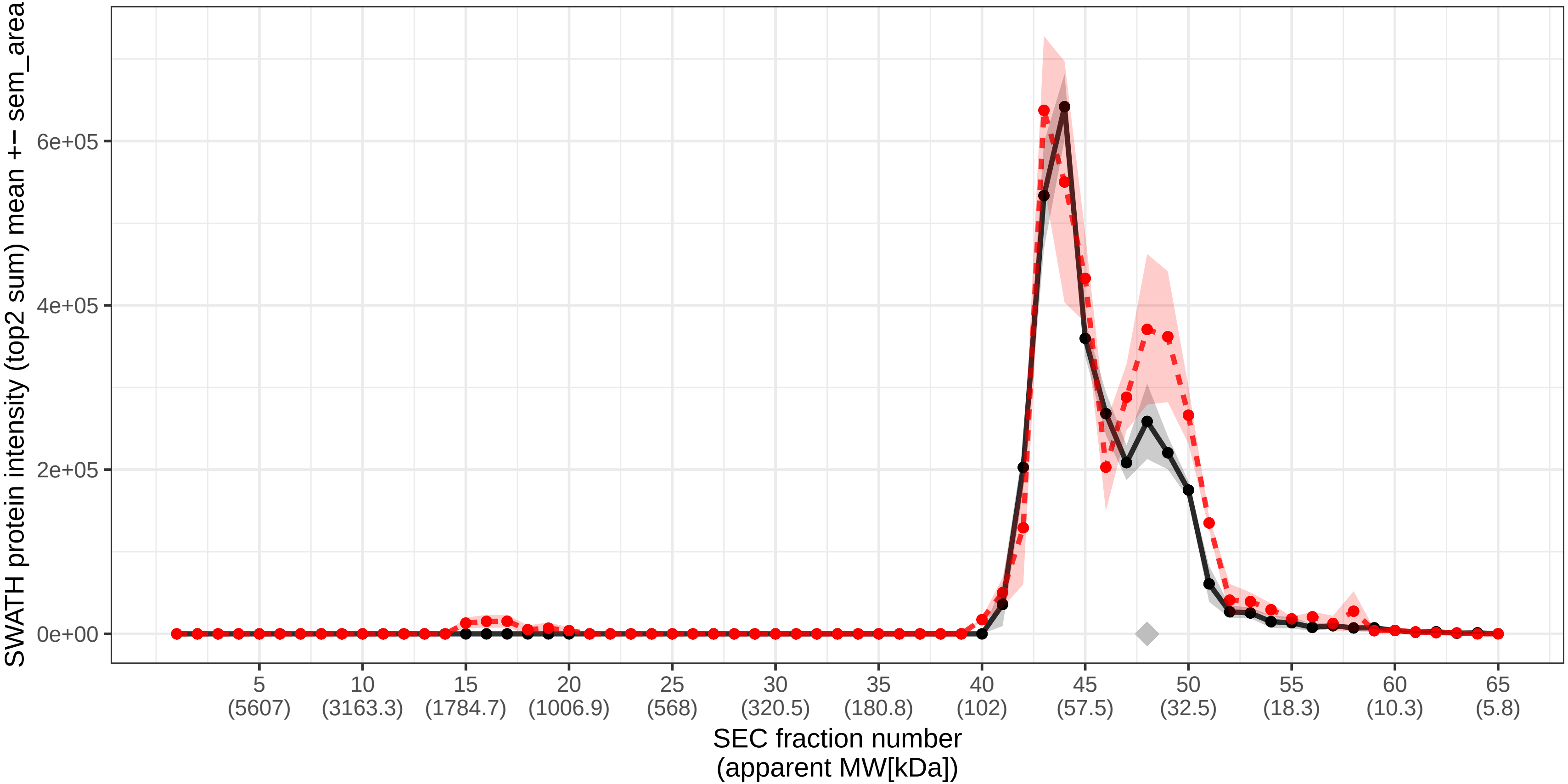

Supplement: Data S1. SEC-SWATH-MS Protein Chromatograms, Related to Figure 1 [file mmc6.zip › SECchrom_Q16539_MK14_HUMAN_MAPK14_CSBP_CSBP1_CSBP2_CSPB1_MX.pdf]
